# Genomic Analysis of *Salmonella enterica* Serovar Typhimurium Definitive Phage Type 104

**DOI:** 10.3201/eid1905.121395

**Published:** 2013-05

**Authors:** Hidemasa Izumiya, Jun Terajima, Shouji Yamamoto, Makoto Ohnishi, Haruo Watanabe, Akemi Kai, Takayuki Kurazono, Masumi Taguchi, Tetsuo Asai, Masato Akiba, Yuko Matsumoto, Yutaka Tamura

**Affiliations:** National Institute of Infectious Diseases, Tokyo, Japan (H. Izumiya, J. Terajima, S. Yamamoto, M. Ohnishi, H. Watanabe);; Tokyo Metropolitan Institute of Public Health, Tokyo (A. Kai); Saitama Institute of Public Health, Saitama, Japan (T. Kurazono);; Osaka Prefectural Institute of Public Health, Osaka, Japan (M. Taguchi);; National Veterinary Assay Laboratory, Tokyo (T. Asai);; National Institute of Animal Health, Ibaraki, Japan (M. Akiba);; Yokohama City Institute of Health, Kanagawa, Japan (Y. Matsumoto);; Rakuno Gakuen University, Hokkaido, Japan (Y. Tamura)

**Keywords:** MLVA, *Salmonella* *enterica* serovar Typhimurium, DT104, phage type, bacteria

**To the Editor:** *Salmonella enterica* is among the leading causes of foodborne diseases worldwide. Multidrug-resistant *S. enterica* serovar Typhimurium definitive phage type (DT) 104 emerged during the early 1990s in the United Kingdom and spread worldwide thereafter (*1*). This phage-type strain harbors a chromosomally encoded genomic island, Salmonella Genomic Island 1, which is typically responsible for resistance to ampicillin, chloramphenicol, streptomycin, sulfonamide, and tetracycline (*2*). Multilocus variable-number tandem-repeat analysis (MLVA) is an established molecular epidemiologic tool; its high-resolution power has been applied to the subtyping of a variety of bacterial species (*3*). An MLVA system has been developed for analyzing *S. enterica* serovar Typhimurium (*4*,*5*).

The design of an MLVA system relies on the analyzed genome sequences. In this study, we found and evaluated a variable-number tandem-repeat region, or locus, designated DT104o. The locus is specific to *S. enterica* ser. Typhimurium DT104, according to the sequence of NCTC 13348 (available from the Sanger Institute, http://www.sanger.ac.uk/resources/downloads/bacteria/salmonella.html). The repeat unit sequence was CTCAGAA/TTCTGAG, spanning 1952121–1952274 on the reference genome or 22 repeats of 7 nt.

We used 266 apparently independent isolates of *S. enterica* serovar Typhimurium collected during 1981–2012; 103 were from human samples and 163 from non-human sources. Bacteriophage typing was performed according to Anderson’s method and scheme (*6*). Types of 100 isolates were in the DT104 group, comprising DT104, DT104B, and U302, the latter being related to DT104 (*2*); MLVA was performed by using the 5 loci (STTR3, STTR5, STTR6, STTR9, and STTR10) with slight modifications (*4*,*5*). The DT104o locus was tested by using primers o-for (5′-GTCAACATGAACTGCCCCTCA-3′), labeled with NED, and o-rev (5′-TTTGCTCTTCGCTCTTAGCAATC-3′); this spanned 1952367–1952043 on the reference sequence, resulting in a 325-bp product with 171-bp offset.

For all 266 isolates tested, the number of alleles and the Simpson’s index of diversity score (*D*) identified in each locus are summarized in the Table. The 5 common and DT104o loci displayed high discriminatory power: DT104o was specific for the DT104 group, and all 100 DT104 group isolates displayed amplified products with 13–40 repeat copy numbers; the others showed the null allele. Focusing only on the 100 DT104 group isolates, the discriminatory power of STTR9 and STTR3 were poor, whereas STTR5, STTR6, STTR10, and DT104o displayed high discriminatory powers (Table). In addition, using the 5 common loci (MLVA5) in analysis, we identified 66 types with a *D* value of 0.974; use of MLVA5 plus the DT104o locus (MLVA6) identified 83 types with a *D* value of 0.984. These results indicate that the DT104o locus is highly specific and therefore useful as an additional molecular epidemiologic marker for analyzing *S. enterica* ser. Typhimurium DT104.

**Table:** Number of alleles and Simpson's index of diversity score in *Salmonella* enterica serovar Typhimurium definitive phage type 104 in humans*

Locus	All, n = 266			DT104-group, n = 100	
	No. alleles†	*D*		No. alleles†	*D*
STTR9	5	0.62		1	0.00
STTR5	17	0.87		10	0.76
STTR6	20	0.93		16	0.90
STTR10	25	0.90		20	0.93
STTR3	12	0.73		2	0.20
DT104o	24	0.60		23	0.92

*D, Simpson’s index of diversity score. †Including the null allele.

Because DT104o was highly variable, 5 DT104 strains were tested for the frequency of variants at each locus after 5 serial passages by using liquid culture: cultures were diluted 1:1,000 at each passage. Sixteen colonies of each strain were tested by using MLVA6 (Technical Appendix Table 1). No variants were observed in STTR3, STTR9, or STTR10. STTR5, STTR6, and DT104o each showed 1 variant of 80 colonies. The results suggest that DT104o would not be less stable than other loci.

We also found that DT104o could provide more discriminatory power to MLVA5 in some settings (Technical Appendix Table 2). We compared 2 settings using isolates from non-human samples. Setting 1 comprised isolates 1a and 1b from an outbreak during 1996 and isolate 1c in 2007. Isolates 1a and 1b were identical by MLVA6. Isolate 1c was identical by MLVA5 but not by MLVA6. In Setting 2, three isolates obtained in different years also were identical by MLVA5, but differed from each other by MLVA6. This suggests that MLVA6 could be useful in some epidemiologic settings such as in an outbreak investigation, though more extensive study would be required to confirm this suggestion.

The DT104o locus is located at the proximal region of fragment 180 comprised of a prophage structure, which was proven to be DT104-specific in a previous study (*7*). This finding is consistent with the results of our study.

In conclusion, development of an MLVA system is dependent upon the genome sequences available, and the system is usually used for molecular subtyping of a certain serotype in a particular organism. However, a specific group of strains could cause a pandemic and become a target of public health concern, as was *S. enterica* ser. Typhimurium DT104. The MLVA system could be improved by adding loci based on the genome sequence of such pandemic strains. In this study, we showed that the newly identified DT104o locus could be useful in identification and subtyping of *S. enterica* ser. Typhimurium DT104.

Technical Appendix Variation of locus and repeat copy number in settings identical in MLVA5 but not in MLVA6 in *Salmonella* *enterica* serovar Typhimurium definitive phage type 104 isolated from nonhuman samples.
