# Supplementary material for: Genomic Analysis of Salmonella enterica Serovar Typhimurium Definitive Phage Type 104
Source: Emerg Infect Dis. 2013 May;19(5):823–4. doi: 10.3201/eid1905.121395 (PMC3647504; doi:10.3201/eid1905.121395)
Supplement: Technical Appendix — Variation of locus and repeat copy number in settings identical in MLVA5 but not in MLVA6 in Salmonella enterica serovar Typhimurium definitive phage type 104 isolated from nonhuman samples. [file 12-1395-Techapp-s1.pdf]

Article DOI: http://dx.doi.org/10.3201/eid1905.121395

# Genomic Analysis of *Salmonella enterica* Serovar Typhimurium Definitive Phage Type 104

## Technical Appendix

Technical Appendix Table 1. Variation of locus in *Salmonella enterica* serovar Typhimurium definitive phage type 104, isolated from human samples, after serial passage

| | Locus | | | | | | | | | |
|---|---|---|---|---|---|---|---|---|---|---|
| | Variant* | | | | | | | | | |
| | STTR9 | STTR5 | | STTR6 | | STTR10 | STTR3 | DT104o | | |
| Strain | 0 | 0 | 1 | 0 | 1 | 0 | 0 | 0 | 1 | SLV† |
| Hd241 | 16 | 15 | 1 | 16 | | 16 | 16 | 16 | | 1 |
| Hd242 | 16 | 16 | | 15 | 1 | 16 | 16 | 16 | | 1 |
| Hd243 | 16 | 16 | | 16 | | 16 | 16 | 16 | | 0 |
| Hd244 | 16 | 16 | | 16 | | 16 | 16 | 15 | 1 | 1 |
| Hd245 | 16 | 16 | | 16 | | 16 | 16 | 16 | | 0 |

Technical Appendix Table 2. Repeat copy number in settings identical in MLVA5 but not in MLVA6 in *Salmonella enterica* serovar Typhimurium definitive phage type 104 isolated from non-human samples

| Setting | Isolate | Year | STTR9 | STTR5 | STTR6 | STTR10 | STTR3 | DT104o |
|---|---|---|---|---|---|---|---|---|
| 1 | 1a* | 1996 | 3 | 13 | 15 | 18 | 0311 | 18 |
| | 1b* | 1996 | 3 | 13 | 15 | 18 | 0311 | 18 |
| | 1c | 2007 | 3 | 13 | 15 | 18 | 0311 | 23 |
| 2 | 2a | 2004 | 3 | 15 | −2† | 28 | 0311 | 13 |
| | 2b | 2007 | 3 | 15 | −2 | 28 | 0311 | 16 |
| | 2c | 2008 | 3 | 15 | −2 | 28 | 0311 | 18 |

*Isolated from the same non-human outbreak. 1b was not included the 266 isolates in the study.

†Null allele.
